# Bioenergetic signatures of neurodevelopmental regression

**DOI:** 10.3389/fphys.2024.1306038

**Published:** 2024-02-19

**Authors:** Richard E. Frye, Patrick J. McCarty, Brianna A. Werner, Shannon Rose, Adrienne C. Scheck

**Affiliations:** ^1^ Autism Discovery and Treatment Foundation, Phoenix, AZ, United States; ^2^ Tulane University School of Medicine, New Orleans, LA, United States; ^3^ Creighton University School of Medicine Phoenix Regional Campus, Phoenix, AZ, United States; ^4^ Arkansas Children’s Research Institute, Little Rock, AR, United States; ^5^ Department of Child Health, University of Arizona College of Medicine—Phoenix, Phoenix, AZ, United States

**Keywords:** autism, mitochondria, sex effect, age effect, neurodevelopemental regression

## Abstract

**Background:** Studies have linked autism spectrum disorder (ASD) to physiological abnormalities including mitochondrial dysfunction. Mitochondrial dysfunction may be linked to a subset of children with ASD who have neurodevelopmental regression (NDR). We have developed a cell model of ASD which demonstrates a unique mitochondrial profile with mitochondrial respiration higher than normal and sensitive to physiological stress. We have previously shown similar mitochondrial profiles in individuals with ASD and NDR.

**Methods:** Twenty-six ASD individuals without a history of NDR (ASD-NoNDR) and 15 ASD individuals with a history of NDR (ASD-NDR) were recruited from 34 families. From these families, 30 mothers, 17 fathers and 5 typically developing (TD) siblings participated. Mitochondrial respiration was measured in peripheral blood mononuclear cells (PBMCs) with the Seahorse 96 XF Analyzer. PBMCs were exposed to various levels of physiological stress for 1 h prior to the assay using 2,3-dimethoxy-1,4-napthoquinone.

**Results:** ASD-NDR children were found to have higher respiratory rates with mitochondria that were more sensitive to physiological stress as compared to ASD-NoNDR children, similar to our cellular model of NDR. Differences in mitochondrial respiration between ASD-NDR and TD siblings were similar to the differences between ASD-NDR and ASD-NoNDR children. Interesting, parents of children with ASD and NDR demonstrated patterns of mitochondrial respiration similar to their children such that parents of children with ASD and NDR demonstrated elevated respiratory rates with mitochondria that were more sensitive to physiological stress. In addition, sex differences were seen in ASD children and parents. Age effects in parents suggested that mitochondria of older parents were more sensitive to physiological stress.

**Conclusion:** This study provides further evidence that children with ASD and NDR may have a unique type of mitochondrial physiology that may make them susceptible to physiological stressors. Identifying these children early in life before NDR occurs and providing treatment to protect mitochondrial physiology may protect children from experiencing NDR. The fact that parents also demonstrate mitochondrial respiration patterns similar to their children implies that this unique change in mitochondrial physiology may be a heritable factor (genetic or epigenetic), a result of shared environment, or both.

## 1 Introduction

Autism spectrum disorder (ASD) is a behaviorally defined neurodevelopmental disorder (American Psychiatric Association, 2013) which continues to increase in prevalence with the current estimated prevalence of 1 in 36 children in the United States (Maenner et al., 2023). Despite decades of investigation, in most cases the cause of ASD remains uncertain. ASD does appear to be associated with disorders in several metabolic pathways, including abnormalities in folate, cobalamin, branched chain amino acids, fatty acid, and mitochondrial metabolism.

Although disorders of mitochondrial metabolism have been implicated in ASD, many times the abnormalities are not well described using the classic criteria for the diagnosis of mitochondrial disease. For example, while the overall prevalence of classical mitochondrial disease in children with ASD is 5%, about 30% of children with ASD have biomarkers of abnormal mitochondrial function (Rossignol and Frye, 2012) and some studies have found that 80% of immune cells from children with ASD have electron transport chain (ETC) complex deficits (Giulivi et al., 2010; Napoli et al., 2014).

Mitochondrial dysfunction may be related to the approximate one-third of children with ASD who experience neurodevelopmental regression (NDR), an enigmatic phenomenon where children develop normally until the second year of life at which time they lose previously acquired skills and develop symptoms of ASD, frequently in the context of a physiological stress such as an illness or seizure. NDR is more common in children with ASD and mitochondrial disease (Rossignol and Frye, 2012), and one study demonstrated that the majority of children with ASD and mitochondrial disease developed ASD symptoms after a sudden rapid NDR associated with a fever (Shoffner et al., 2010). This is consistent with other studies which have shown a characteristic NDR following illness in children with mitochondrial disease (Edmonds et al., 2002). Thus, it is possible that children with ASD and NDR might have underlying mitochondrial dysfunction which might be triggered by a physiological stress.

To better understand mitochondrial abnormalities in ASD we previously developed an *in vitro* cell model and an assay called the Mitochondrial Oxidative Stress Test (MOST). The purpose of the MOST is to systematically investigate changes in mitochondrial function in the context of physiological stress. We hypothesized that a subset of children with ASD would have mitochondria which were more vulnerable to physiological stress. Using the Seahorse 96 XF analyzer, indices of mitochondrial respiration were measured in control and ASD lymphoblastic cell lines (LCLs) at baseline and after systematic physiological stress with 2,3-dimethoxy-1,4-napthoquinone (DMNQ).

Using the MOST, we found that the LCLs from about one-third of children with ASD were more vulnerable to physiological stress. Important respiratory parameters such as reserve capacity (RC), a measure of mitochondrial health, quickly plummeted in these cells when challenged with physiological stress as compared to other cell lines (Rose et al., 2014). These physiologically vulnerable cell lines are called AD-A (ASD with abnormal mitochondrial function). Mitochondria from the remaining ASD LCLs were essential equivalent to controls and then are called AD-N (ASD with normal mitochondrial function). Interestingly, at baseline (i.e., without physiological stress) the AD-A LCLs demonstrate respiratory rates approximately twice that of the AD-N and healthy control LCLs. We have replicated these results in LCLs in seven studies (Rose et al., 2014; Rose et al., 2015a; Frye et al., 2016; Frye et al., 2017; Rose et al., 2017; Rose et al., 2018a; Bennuri et al., 2019).

To further investigate whether this pattern of atypical mitochondrial respiration was related to NDR, we previously measured mitochondrial respiration in peripheral blood mononuclear cells (PBMCs) from individuals with ASD. ASD patients with NDR showed significantly elevated mitochondrial respiration at baseline, just like our LCL model (Singh et al., 2020). However, in the PBMC study we did not use the MOST to investigate whether PBMCs from those with ASD and NDR also showed the sensitivity to physiological stress that is seen in the AD-A LCLs.

Thus, to further investigate these unique changes in mitochondrial function potentially related to NDR in ASD, this study extends our previous work in several ways:

1. We validate the findings in our previous PBMC study in a new cohort. We hypothesize that the PBMCs from children with ASD and NDR will have elevated respiratory rates, just like our previous study. Replicating novel findings is always important in scientific research in order to validate previous discoveries.
2. The MOST used in LCL studies is adapted for use in PBMCs. We hypothesize that DMNQ can physiologically stress PBMCs similar to the LCL assay. Data is presented to validate that DMNQ causes physiological stress to the mitochondria in PBMCs.
3. PBMCs from the new cohort are physiologically stressed using the MOST to determine if they have a similar response to physiological stress as our LCL model. We hypothesize that mitochondrial respiration in PBMCs from children with ASD and NDR will show both elevated respiratory rates and greater sensitivity to physiological stress, parallel to our *in vitro* AD-A LCL cell model. Finding that individuals with ASD and NDR show mitochondrial abnormalities like the AD-A LCLs would strengthen the evidence that the LCL model represents mitochondrial dysfunction in ASD and that mitochondrial dysfunction is related to NDR.
4. A previous LCL study found that abnormalities in mitochondrial respiration were unique to ASD as compared to typically developing (TD) siblings (Rose et al., 2017). Thus, we hypothesis that abnormalities in mitochondrial respiration will be unique to those with ASD and NDR and will not be shared by their TD siblings. Understanding whether TD siblings share mitochondrial respiratory abnormalities will provide support for the uniqueness of mitochondrial respiratory abnormalities in ASD and help determine whether there might be matroclinous inheritance since ASD siblings also inherit maternal mitochondria.
5. To further investigate the possibility of matroclinous inheritance, transgenerational aspects of mitochondrial respiration were investigated by measuring mitochondrial respiration in both mothers and fathers of the children with ASD. If the respiratory abnormalities seen in children with ASD and NDR are found in their mothers but not fathers, it would suggest matroclinous inheritance and that the respiratory abnormalities are innate to inherited mitochondria.

## 2 Methods

### 2.1 Participants

Protocols were approved by the Institutional Review Board at Phoenix Children’s Hospital (Phoenix, AZ). After data collection, all data and specimens were deidentified. Exclusion criteria were 1) chronic treatment with medications that would detrimentally affect mitochondrial function such as antipsychotic medications; 2) vitamin or mineral supplementation exceeding the recommended daily allowance, and 3) prematurity (<36 weeks gestation). Inclusion criteria for this study included a diagnosis of ASD or being a relative of an individual with ASD. The ASD diagnosis was defined by one of the following: 1) a gold-standard diagnostic instrument: the Autism Diagnostic Observation Schedule or Autism Diagnostic Interview-Revised and/or 2) Diagnostic Statistical Manual (DSM) diagnosis by a physician, along with standardized validated questionnaires and diagnosis confirmation by the principal investigator (REF). We have validated the latter criteria by showing that participants defined by this criterion are well within the diagnostic criteria for ASD using the Autism Diagnostic Interview-Revised (Frye et al., 2018).

The NDR history was obtained using the Developmental and Neurobehavioral Regression (DANR) questionnaire which has been developed as part of our ASD research program and used in our previous studies (Frye et al., 2021a; Frye et al., 2022). The DANR records detailed information about NDR including specific questions on premorbid functioning before the regression, duration of the regression, specific skills lost and when the skills were regained, whether there was a single or multiple regressions and any known trigger such as illness, fever or seizure.

Parents of participants provided written informed consent. Children underwent a fasting blood draw in the morning. Control individuals did not have any neurological disorders, developmental delays or mitochondrial disease.

### 2.2 Behavioral measurements

Parents completed the aberrant behavior checklist (ABC) and the social responsiveness scale (SRS) questionnaire. These are common validated measures for providing an assessment of ASD symptoms in children with ASD (Delhey et al., 2017; Frye et al., 2018). The ABC is a 58-item questionnaire that measures disruptive behaviors and has convergent and divergent validity. The SRS is a 65-item questionnaire that measures the severity of ASD symptoms, including social skill deficits and repetitive behaviors across five domains. The SRS has been shown to have a good correlation to gold-standard instruments.

### 2.3 Blood collection and processing

Up to 20 mL of blood was collected into an ethylenediaminetetraacetic acid (EDTA)-Vacutainer tube, chilled on ice and centrifuged at 1500 *g* for 10 min at 4°C to separate plasma within 30 min of collection. Just prior to the Seahorse assay, plasma was removed and replaced with room temperature wash buffer containing Ca^+2^/Mg^+2^-free PBS with 0.1% bovine serum albumin (BSA) and 2 mM EDTA. Diluted blood was then layered on top of Histopaque-1077 (Sigma Aldrich, St. Louis, MO, United States) and centrifuged at 400 g for 30 min at room temperature. PBMCs were removed from the interface and were washed twice with wash buffer, the viable cells were counted using trypan blue exclusion and a Countess II Automated Cell Counter (Invitrogen, ThermoFisher Scientific, Waltham, MA). The isolation procedure duration was 90–120 min. Our recent study demonstrated that performing the Seahorse assay within 4 h s of collection using EDTA collection tubes provides the most optimal results (Werner et al., 2022). Thus, the Seahorse assay was performed well within this time frame.

### 2.4 Mitochondrial respiration assay

PBMCs were placed in assay media (unbuffered RPMI without phenol red; supplemented with 1 mM pyruvate, 2 mM glutamate and 25 mM glucose) that was warmed to 37°C and pH adjusted to 7.4 prior to cell suspension. XFe96 plates (Agilent Technologies, Santa Clara, CA) were prepared by adding 25 μL of 50 μg/mL poly-D-lysine (EMD Millipore, Burlington, MA) to each well for 2 hours, washing with 250 μL sterile water and drying in a laminar flow hood overnight prior to seeding with 4 × 10^5^ viable PBMCs per well. After seeding, the plates were spun with slow acceleration (4 on a scale of 9) to a maximum of 100 g for 2 min and then allowed to stop with zero braking (Eppendorf Model 5810R Centrifuge, ThermoFisher Scientific). The plate orientation was reversed, and the plate was spun again in the same fashion. Prior to the Seahorse assay, XFe96 wells were visualized using an inverted microscope to ensure PBMCs were evenly distributed in a single layer. For each experimental condition, at least four replicate wells were measured simultaneously to improve assay reliability. Runs with clear measurement probe failure, reagent injection failures, or non-physiology measurements (ATP-linked respiration (ALR) or proton-leak respiration (PLR) < −1 pmol/min) were eliminated.

The Seahorse 96 XF Analyzer simultaneously measures oxygen consumption rate (OCR) and extracellular acidification rate (ECAR) in real-time using a 96-well plate and a wide range of intact living cell types (Hill et al., 2010; Perez et al., 2010). We have used the Seahorse to assess mitochondrial abnormalities related to ASD using LCLs (Rose et al., 2014; Rose et al., 2015a; Rose et al., 2015b; Frye et al., 2016; Frye et al., 2017; Rose et al., 2017; Rose et al., 2018a; Bennuri et al., 2019) as well as PBMCs (Burger et al., 2017) and have linked variation in mitochondrial function in PBMCs to immune abnormalities (Jyonouchi et al., 2019) and prenatal environmental exposures (Frye et al., 2020, 2021a).

**FIGURE 1:**
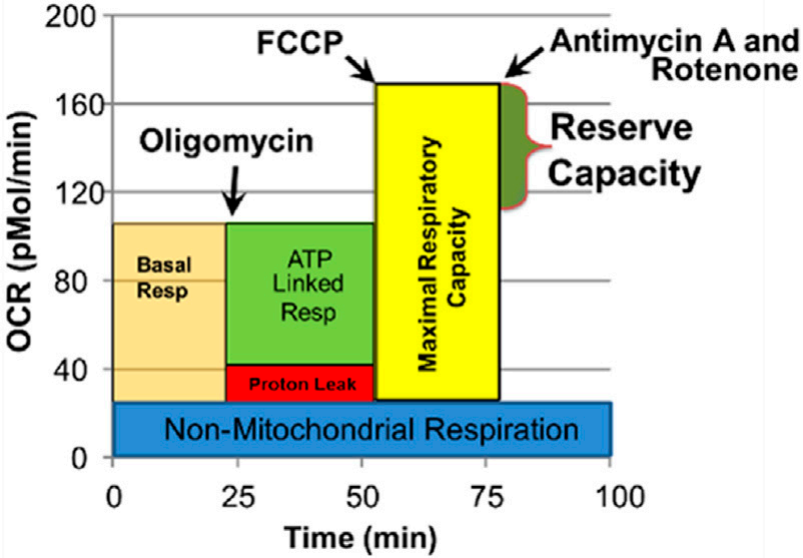
Seahorse assay and various mitochondrial respiratory parameters. Oxygen consumption rate (OCR) is measured to determine mitochondrial activity. Three OCRs are measured over 18 min to determine mitochondrial activity for each segment of the assay. Regents are added to determine parameters of mitochondrial activity. Basal Respiration is the difference between baseline OCR and non-mitochondrial OCR. Oligomycin, which is a complex V inhibitor, is added to determine the portion of Basal Respiration that is ATP-Linked Respiration and Proton-Leak Respiration. Carbonyl cyanide-p-trifluoromethoxyphenyl-hydrazon (FCCP), a protonophore, is added to collapse the inner membrane gradient, driving the mitochondria to respire at their maximal rate. This determines Maximal Respiratory Capacity. Antimycin A and Rotenone, complex III and I inhibitors, stop mitochondrial respiration to determine the non-mitochondrial respiration. Reserve Capacity is the difference between Basal Respiration and Maximal Respiratory Capacity. Reprinted with permission from Frye et al. (2021a), licensed under CC BY 4.0.

The Seahorse assay is a 4-step process which monitors OCR three times during four distinct periods in response to various reagents that activate or inhibit the ETC (Figure 1). Several key parameters are derived from this process. We have recently examined the reliability of the Seahorse assay, finding excellent intraclass correlation coefficients across parameters for measures in PBMCs (Frye et al., 2021a).

#### 2.4.1 Assay measurements

1. Baseline OCR/ECAR: Baseline measurement before introducing any reagents.
2. OCR/ECAR after oligomycin: Oligomycin is a complex V inhibitor which shuts down the production of ATP so the OCR related to ATP production can be determined and maximum ECAR, which represents the utilization of glucose, can be determined.
3. Maximal OCR: Carbonyl cyanide-p-trifluoromethoxyphenyl-hydrazone (FCCP) is used to collapse the mitochondrial inner membrane gradient, inducing the mitochondria to function at the maximum extent possible.
4. Antimycin A and Rotenone are added to shut down ETC complex activity to measure OCR from non-ETC processes.

#### 2.4.2 Mitochondrial function metrics

1. ATP-Linked Respiration (ALR): OCR is attributed to ATP production. ATP-Linked respiration = Baseline OCR - OCR after oligomycin.
2. Proton-Leak Respiration (PLR): The amount of OCR that is associated with protons leaking through the inner mitochondrial membrane in order to control oxidative stress. Proton-Leak Respiration = OCR after oligomycin - Non-Mitochondrial OCR.
3. Maximal Respiratory Capacity (MRC): The maximum respiratory rate of the ETC. This parameter is thought to be sensitive to deficits in mitochondrial biogenesis, mtDNA damage and/or inhibition of ETC function. Maximal Respiratory Capacity = Maximal OCR - Non-Mitochondrial OCR.
4. Reserve Capacity (RC): The increase is respiration produced by oxidative phosphorylation when there is a sudden increase in energy demand. This parameter is an index of mitochondrial health; when it becomes negative, the mitochondrion is unhealthy, leading the cell towards apoptosis. Reserve Capacity = Maximal OCR - Baseline OCR.
5. Glycolytic Rate (GR): The rate of glucose utilization at baseline. Glycolytic Rate = baseline ECAR.
6. Glycolytic Reserve Capacity (GRC): The amount of extra capacity of glycolysis when oxidative phosphorylation cannot produce cellular energy. Glycolytic Reserve Capacity = ECAR after oligomycin—Glycolytic Rate.

### 2.5 Redox challenge

Similar to our previous studies on LCLs (Rose et al., 2014; Rose et al., 2015a; Frye et al., 2016; Frye et al., 2017; Rose et al., 2017; Rose et al., 2018a; Bennuri et al., 2019), PBMCs were exposed to various concentrations of DMNQ (Sigma-Aldrich, St. Louis, MO, United States) for 1 h at 37°C in a non-CO_2_ incubator prior to the Seahorse assay. A 5 mg/mL DMNQ solution was diluted in DMEM XF assay media (Agilent Technologies) into a 10X stock and added to cells in an XF-PS plate (Rose et al., 2014). To verify that mitochondrial reactive oxygen species (ROS) increased as DMNQ concentration increased, mitochondrial superoxide was imaged in PBMCs with increasing DMNQ. MitoSox™ Red (Molecular Probes, Inc., Eugene, OR) was used to depict mitochondrial superoxide production using conditions specified by the manufacturer. MitoSox™ fluorescence was visualized after a 15 min incubation with 0–10 µM DMNQ. Nuclei were counterstained with Hoeschst 33342.

### 2.6 Statistical analysis

Analyses were performed using PASW Statistics version 28.0.0.0 (IBM SPSS Statistics, Armonk, NY). Graphs were produced using Excel version 14.0 (Microsoft Corp, Redmond, WA). An alpha of 5% was used as a cutoff for significance.

To analyze the difference in ASD participant characteristics, pearson chi-square test was calculated on cross tabulation tables for sex and race/ethnicity. For continuous variables a multivariate generalized linear model was used to perform an analysis of variance. The model contained NDR status, sex or age factors, where applicable.

Mitochondrial measures were obtained in at least quadruplicate and across several DMNQ concentrations. To account for the repeated measures, a mixed-model linear analysis was performed. This analysis takes into account both within-subject variation from all of the repeated mitochondrial measurements (repeated measurements at each DMNQ concentration and different DMNQ concentrations) on the same individual and the between-subject variation from experimental variables. Interactions were considered where appropriate and the final models were simplified to eliminate any non-significant higher order interactions. Participant characteristics including sex and age were initially added to the models. Age was not significant for the ASD participants and was subsequently eliminated from that model. In general, an alpha of 0.05 was used. Separate sections describe the group, sex and age effects.

The model design is essentially a repeated measures analysis of variance. Power analysis was conducted with G*Power (Kiel, Germany). The power analysis assumes approximately 25 measures per participant with a correlation among repeated measurements of 0.9, alpha = 0.05 and a nonsphericity correction ϵ = 1.0. All analyses contained 4 group comparisons and the smallest analysis had an N = 31. This achieved a power of 99% for finding a small effect (f = 0.10). Thus, all analyses are appropriately powered.

## 3 Results

### 3.1 Adaptation of the mitochondrial oxidative stress test for PBMCs

**FIGURE 2:**
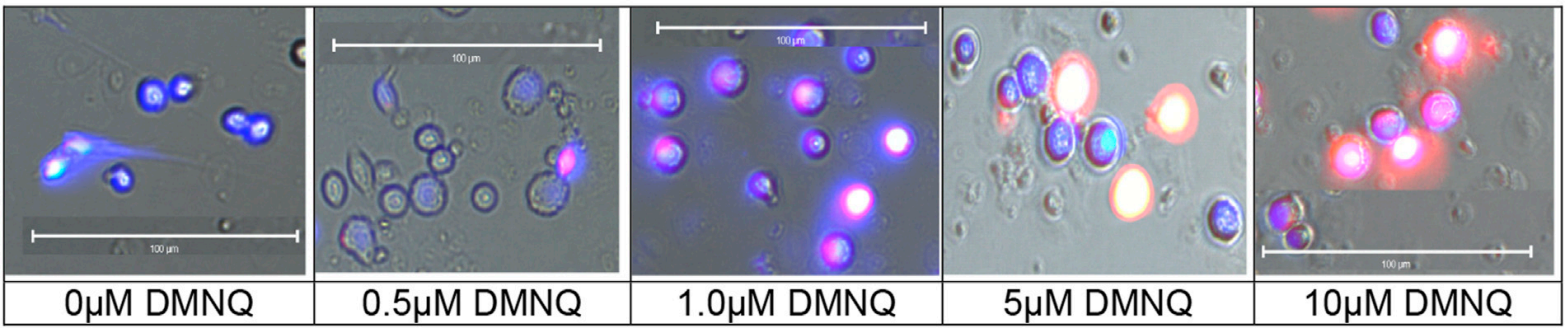
Mitochondrial superoxide (MitoSox™ Red) increases in PBMCs as DMNQ concentration increases (incubated for 15 min). Nuclei were counterstained with Hoeschst 33342 (blue).

The MOST was originally developed with LCLs using DMNQ concentrations of 0.0 μM, 5.0 μM, 10.0 μM, 12.5 μM, and 15.0 μM. To investigate the effects of physiological stress on PBMC, the titration was expanded to include higher and lower DMNQ concentrations. These included 0.0 μM, 0.025 μM, 0.05 μM, 0.1 μM, 0.25 μM, 0.5 μM, 1.0 μM, 2.5 μM, 5.0 μM, 10.0 μM, 15.0 μM, and 20.0 μM. The goal of the titration was to provide enough physiological stress that RC and MRC are driven down towards zero. These DMNQ concentrations caused the desired effect for the respiratory curves. To verify that DMNQ was resulting in mitochondrial physiological stress, mitochondrial superoxide was imaged (Figure 2). As can be seen in Figure 2, mitochondrial superoxide (red color) increased as DMNQ was added as predicted.

### 3.2 Participants

Twenty-six ASD children without a history of NDR (ASD-NoNDR) and 15 ASD children with a history of NDR (ASD-NDR) were recruited from 34 families. Four families had two siblings with ASD while another family had 3 siblings with ASD. In these families, 30 mothers, 17 fathers and 5 typically developing siblings participated. In one family, the NDR status of the children were mixed, so the NDR group of the parents could not be determined and the parents could not be included in the study.

As seen in Table 1, the ASD-NDR group was, on average, older and contained more females than the ASD-NoNDR group, but these differences were not significant. ASD females [mean (SD) age = 18.1 (7.52)] were significantly older than ASD males [mean (SD) age = 10.6 (6.9); F (1, 36) = 5.14, *p* < 0.05]. Although the TD siblings were slightly older than the ASD participants, there was no significant difference in the age. NDR siblings were of similar age to NoNDR siblings. NoNDR parents were significantly younger than the ASD-NDR parents [F (1, 42) = 4.83, *p* < 0.05] and fathers [mean (SD) age = 47.4 (13.9) years] were older than mothers [mean (SD) age = 40.7 (11.4) years; F (1, 42) = 34.35, *p* < 0.001]. Sex and ethnic distribution were similar.

**TABLE 1:** Demographic differences across participant groups.

Clinical group	ASD		Siblings		Parents^a^	
Regression Group	NoNDR^b^	NDR^c^	NoNDR^b^	NDR^c^	NoNDR^b^	NDR^c^
N	26	15	3	2	26	19
Age [Mean (SD)] yrs	10.4 (6.4)	15.3 (9.1)	15.1 (0.5)	16.7 (0)	36.1 (9.0)	52.6 (10.5)
Age [Range] yrs	3.4–30.5	2.8–33.9	14.5–15.6	16.7	30.1–45.4	44.1–64.1
Male	88%	60%	100%	0%	35%	37%
White	77%	100%	66%	100%	77%	100%
Hispanic	19%	0%	33%	0%	19%	0%
African American	4%	0%	0%	0%	4%	0%

^a^ This only included parents that could be included in the study.

^b^ NoNDR: No history of neurodevelopmental regression in the ASD, proband.

^c^ History of neurodevelopmental regression in the ASD, proband.

As seen in Table 2, ASD-NDR participants demonstrated significantly lower social withdrawal, hyperactivity and inappropriate speech on the ABC. Males [mean (SD) = 8.0 (5.3)] demonstrated less stereotyped behavior than females [mean (SD) = 13.7 (7.8) F (1, 36) = 7.11, *p* = 0.01].

**TABLE 2:** Behavioral and autism symptom severity by neurodevelopmental regression status.

Aberrant behavior checklist	^a^ASD-NoNDR [N = 24]	^b^ASD-NDR [N = 15]
Irritability	21.5 (11.5)	15.4 (12.3)
Social Withdrawal	20.5 (8.1)	9.5 (6.1)***
Stereotype Movements	9.3 (5.2)	9.2 (8.0)
Hyperactivity	28.5 (14.4)	13.7 (9.5)**
Inappropriate Speech	6.3 (5.4)	2.0 (1.9)**
Total	72.6 (41.2)	55.4 (30.1)

Social Responsiveness Scale		
Awareness	68.9 (14.6)	67.0 (12.0)
Cognition	72.8 (12.0)	69.8 (11.1)
Communication	75.7 (10.9)	66.7 (13.6)**
Motivation	72.2 (14.6)	67.3 (11.0)
Mannerism	75.6 (13.8)	73.1 (11.7)
Total	77.5 (11.3)	75.1 (11.7)

*p* ≤ 0.05 *; *p* ≤ 0.01 **; *p* ≤ 0.001 ***.

a ASD without a history of neurodevelopmental regression.

b ASD children with a history of neurodevelopmental regression in the ASD proband

The majority of the SRS scores were in the moderate range [t-score 66–75] with SRS communication being better in the ASD-NDR group. Males [mean (SD) = 69.9 (11.2)] demonstrated better SRS cognition [F (1, 36) = 6.69, *p* = 0.01] than females [mean (SD) = 78.2 (10.1)] and males [mean (SD) = 70.4 (13.4)] demonstrated better communication [F (1,36) = 8.18, *p* > 0.01] than females [mean (SD) = 78.4 (7.4)].

### 3.3 Comparisons of neurodevelopmental regression groups

The group comparisons between children with ASD with and without NDR, between children with ASD and their siblings and between parents with children with ASD with and without NDR are presented in separate sections.

#### 3.3.1 Children with regressive vs. non-regressive ASD

In our LCL model of ASD-NDR, LCLs with mitochondrial dysfunction (AD-A) demonstrated respiratory rates twice that of both controls and ASD LCLs with normal mitochondrial function (AD-N) and showed a greater decrease in MRC and RC as physiological stress was added. Thus, we hypothesized that the ASD-NDR would have higher respiratory rates than ASD-NoNDR individuals. This is represented statistically by the significant mean difference between groups. We also hypothesized that MRC and RC would decrease more as physiological stress was added with the addition of DMNQ as compared to ASD-NoNDR individuals. This is represented statistically by the significant interaction between the group and the slope of the curve. Statistical values are given in Supplemental Table S1.

**FIGURE 3:**
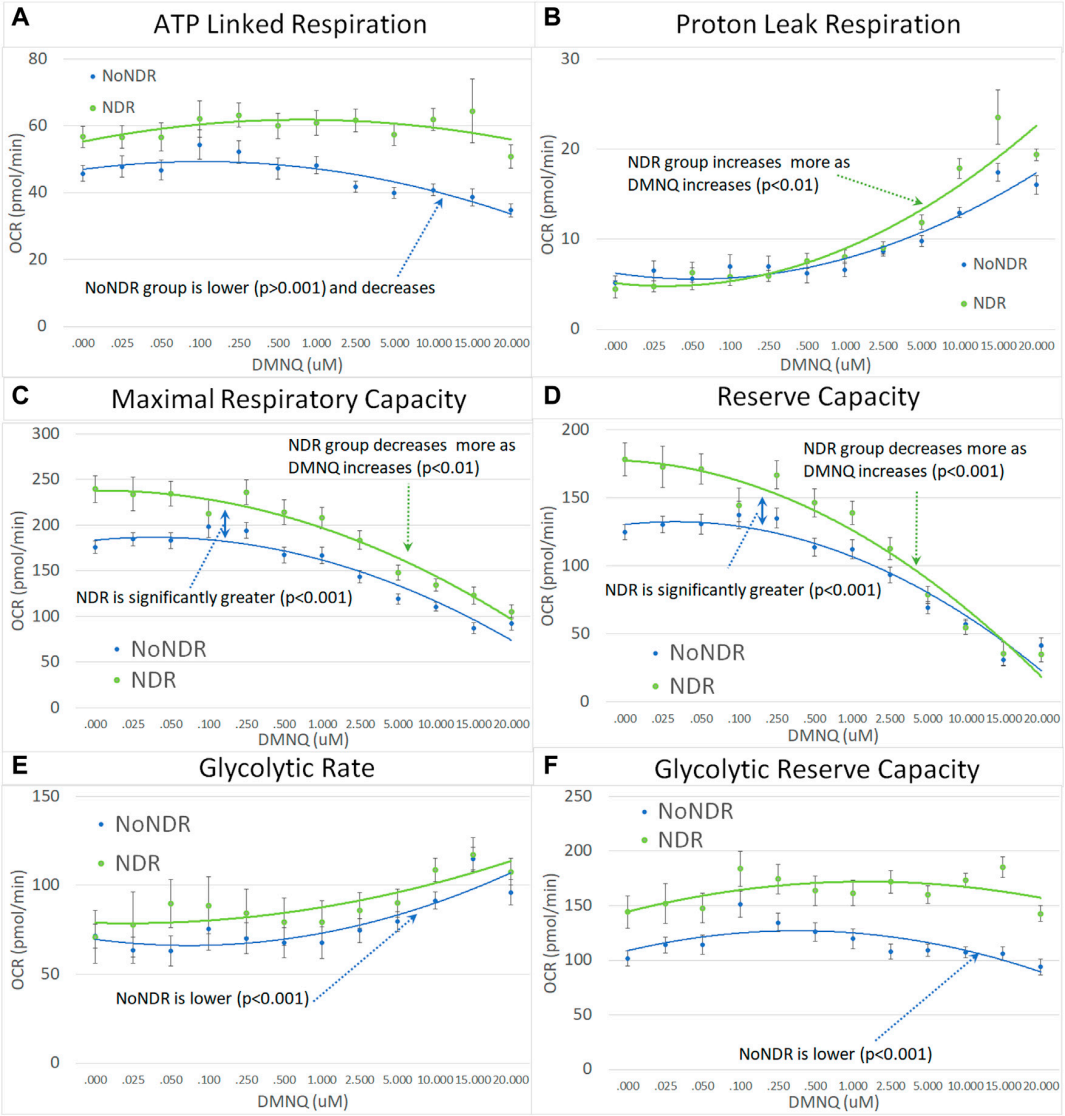
Children with ASD with (NDR) and without (NoNDR) neurodevelopmental regression across mitochondrial respiratory parameters [measured by oxygen consumption rate (OCR)] with various amounts of physiological stress using increasing concentrations of 2,3-dimethoxy-1,4-napthoquinone (DMNQ). In general, findings parallel our previously reported cell model of ASD with neurodevelopmental regression (see text). Standard error bars are shown.

As seen in Figure 3, ASD-NDR individuals demonstrated a significantly higher ALR, MRC and RC (Figures 3A,C,D) as compared to ASD-NoNDR individuals, similar to the AD-A LCLs in our cell model (Rose et al., 2014; Rose et al., 2015a) and similar to our initial PBMC study (Singh et al., 2020). ALR showed less of a decrease in ASD-NDR individuals as compared to ASD-NoNDR individuals as DMNQ increased.

**FIGURE 4:**
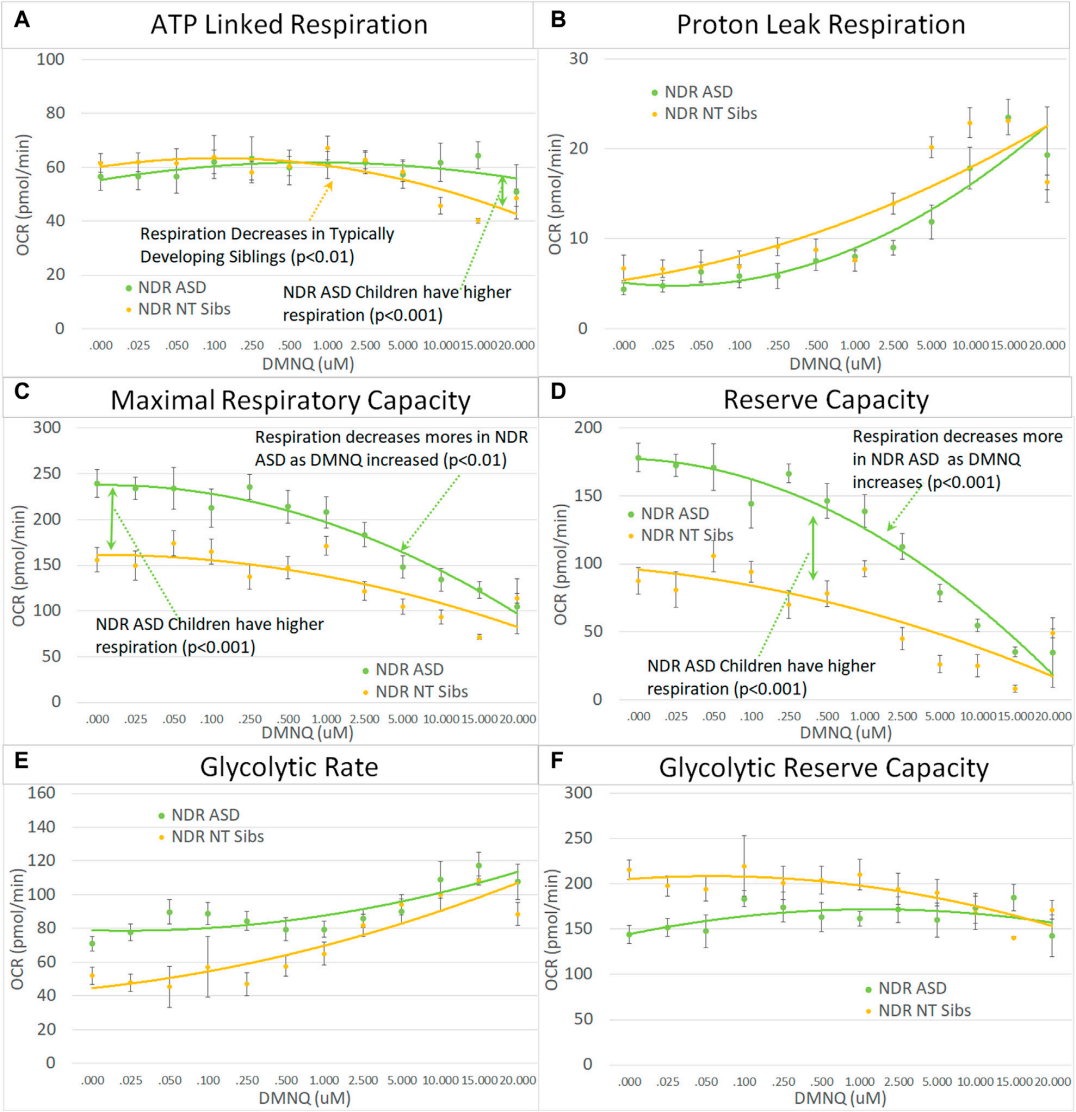
Children with ASD with neurodevelopmental regression (NDR) as compared to their neurotypical (NT) siblings across mitochondrial respiratory parameters [measured by oxygen consumption rate (OCR)] with various amounts of physiological stress using increasing concentrations of 2,3-dimethoxy-1,4-napthoquinone (DMNQ). In general, findings parallel our previously reported cell model of ASD with neurodevelopmental regression (see text). Standard error bars are shown.

Also consistent with our LCL model as DMNQ was increased, PLR showed a greater increase and MRC and RC showed a greater decrease (Figures 4B–D) in ASD-NDR PBMCs as compared to ASD-NoNDR PBMCs (Rose et al., 2014; Rose et al., 2015a). Interestingly, GR and GRC, parameters that were not measured in previous studies comparing AD-A and AD-N LCLs were higher in ASD-NDR as compared to ASD-NoNDR.

#### 3.3.2 Analysis of ASD vs. TD siblings by neurodevelopmental regression group

Next we examined the differences between children with ASD with and without NDR as compared to their TD siblings. Based on our previous studies, we hypothesized that ASD-NDR individuals would demonstrate a different pattern of mitochondrial respiration as compared to their TD siblings, whereas ASD-NoNDR individuals would have respiratory profiles similar to TD siblings. Statistically this is represented by the interaction between participant type (ASD vs. sibling) and NDR status. This interaction can be present in the overall mean and/or the slope of the change in respiration with increasing *in vitro* physiological stress (i.e., increased DMNQ). Statistical values are given in Supplemental Table S2. Only the graphs for NDR participants and their TD siblings are in the main text. Graphs for NoNDR participants are provided in Supplemental Figure S1.

All respiratory measures demonstrated a NDR (NDR vs. NoNDR) by ASD (ASD vs. TD Sibling) interaction, demonstrating that differences in the respiratory curves between individual with ASD and their siblings was dependent on whether the individual with ASD experienced NDR or NoNDR (Figure 4). Consistent with our LCL model (Rose et al., 2014; Rose et al., 2015a) and initial PBMC study (Singh et al., 2020), ASD-NDR participants demonstrated a higher ALR, MRC and RC as compared to TD siblings while these respiratory parameters were similar between ASD and TD siblings for the ASD-NoNDR group. In fact, ASD-NDR individuals had an overall higher MRC and RC without physiological stress (i.e., DMNQ 0), twice that of TD siblings. With increasing DMNQ, MRC and RC decreased rapidly such that is it equal to siblings at maximal physiological stress (i.e., DMNQ 20 µM), consistent with our LCL model. ALR did not change much with increasing DMNQ for ASD-NDR individuals but decreased with increasing DMNQ for TD siblings.

Interestingly, ASD-NoNDR individuals demonstrated lower PLR, GR and GRC values as compared to TD siblings while ASD-NDR individuals demonstrated similar mean values as compared to siblings (Supplementary Figure S1). For ASD-NoNDR participants, PLR did not increase as much as the other groups with increasing DMNQ. Without any physiological oxidative stress, all groups started out with a similar PLR, demonstrating that a stress challenge is needed to uncover this difference. For TD siblings of ASD-NoNDR participants, GR increased more and GRC decreased more with increasing DMNQ as compared to as ASD-NoNDR participants.

#### 3.3.3 NDR vs. NoNDR parents

**FIGURE 5:**
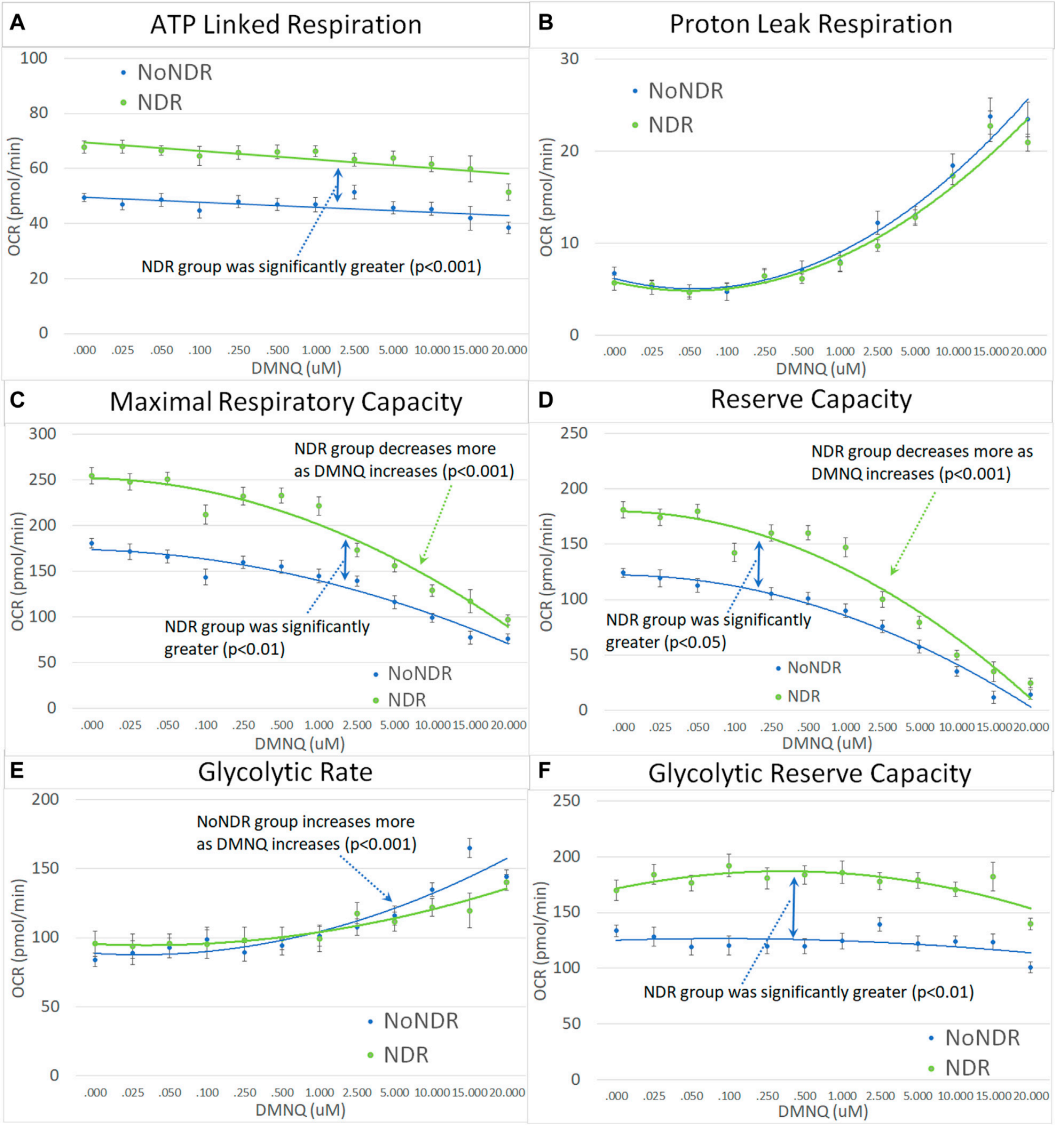
Parents of the children with ASD with (NDR) and without (NoNDR) neurodevelopmental regression across mitochondrial respiratory parameters [measured by oxygen consumption rate (OCR)] with various amounts of physiological stress using increasing concentrations of 2,3-dimethoxy-1,4-napthoquinone (DMNQ). Standard error bars are shown.

Like their children, ASD-NDR parents demonstrated significantly higher ALR, MRC, RC, and GRC as compared to ASD-NoNDR parents (Figures 5A,C,D,F). In addition, like their children, ASD-NDR parents demonstrated a significantly greater decrease in MRC and RC with increasing DMNQ as compared to ASD-NDR parents (Figures 5C,D). In addition, ASD-NDR parents demonstrated less of an increase in GR with increasing DMNQ stress as compared to ASD-NoNDR parents (Figure 5E).

### 3.4 Sex effects

**FIGURE 6:**
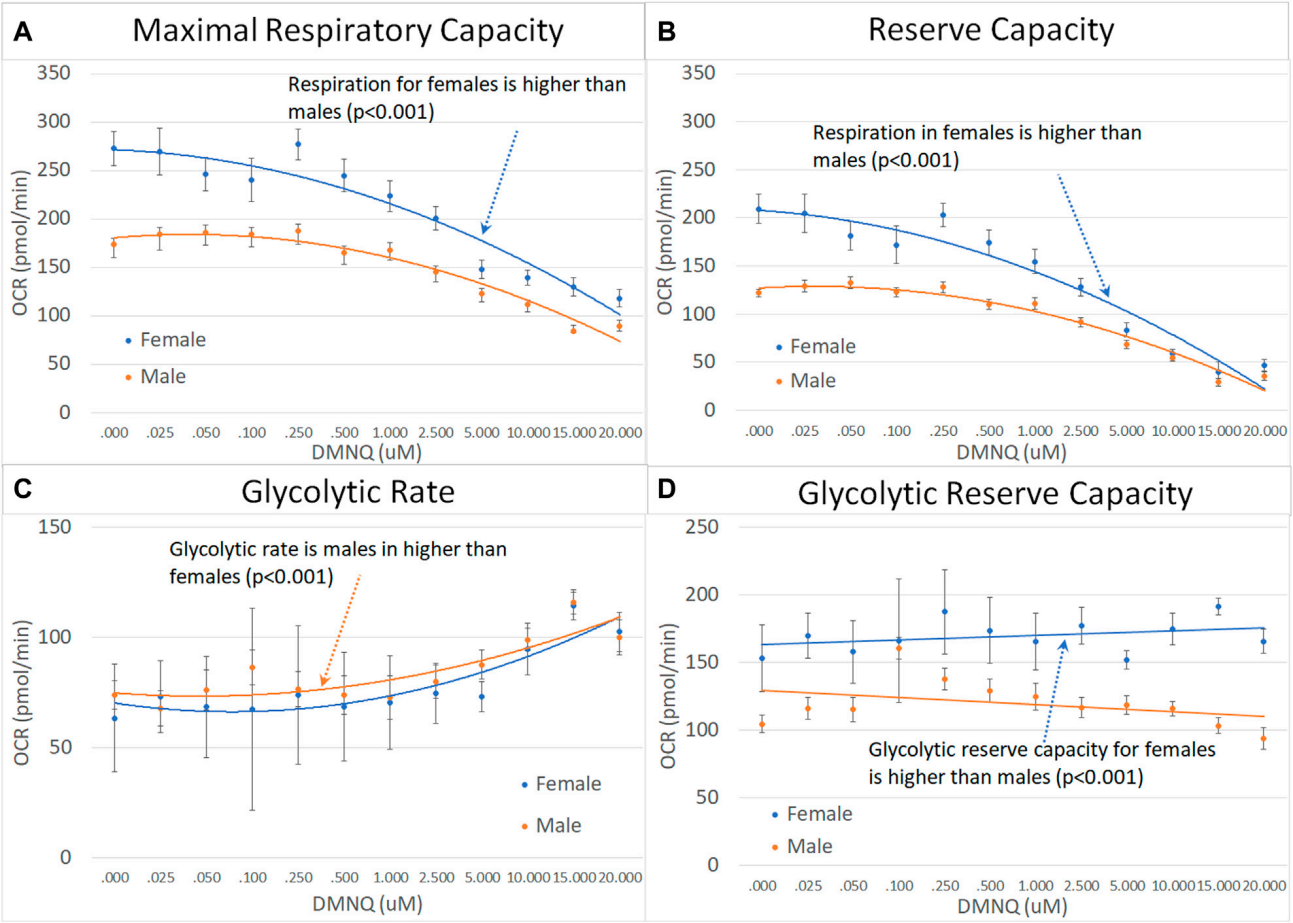
Male and female children with ASD across mitochondrial respiratory parameters [measured by oxygen consumption rate (OCR)] with various amounts of physiological stress using increasing concentrations of 2,3-dimethoxy-1,4-napthoquinone (DMNQ). Standard error bars are shown.

For children with ASD, the MRC, RC, and GRC were significantly higher in females as compared to males and GR was significantly higher in males as compared to females (Figure 6).

**FIGURE 7:**
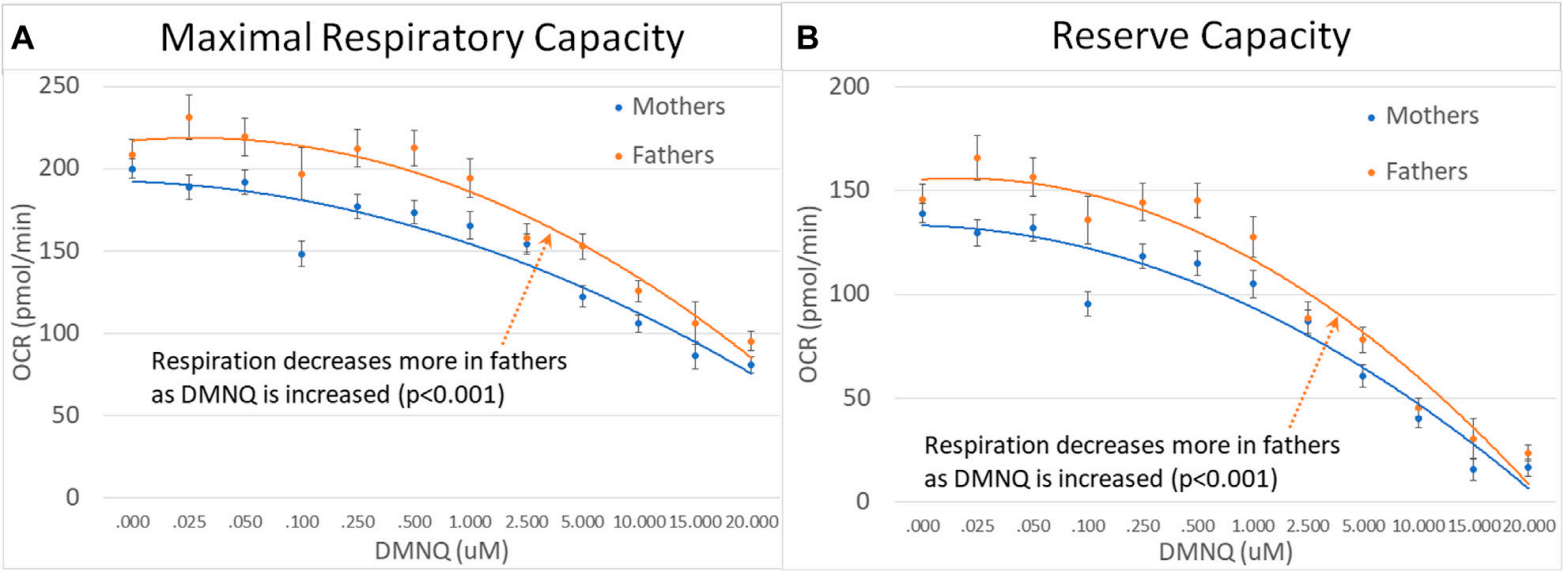
Mothers and fathers of children with ASD across mitochondrial respiratory parameters [measured by oxygen consumption rate (OCR)] with various amounts of physiological stress using increasing concentrations of 2,3-dimethoxy-1,4-napthoquinone (DMNQ). Standard error bars are shown.

For parents of children with ASD, fathers demonstrated a greater decrease in MRC and RC as DMNQ was increased as compared to females (Figures 7A,B), suggesting that mitochondria in male PBMCs were less resilient than mitochondrial in female PBMCs.

### 3.5 Age effects: parental age

**FIGURE 8:**
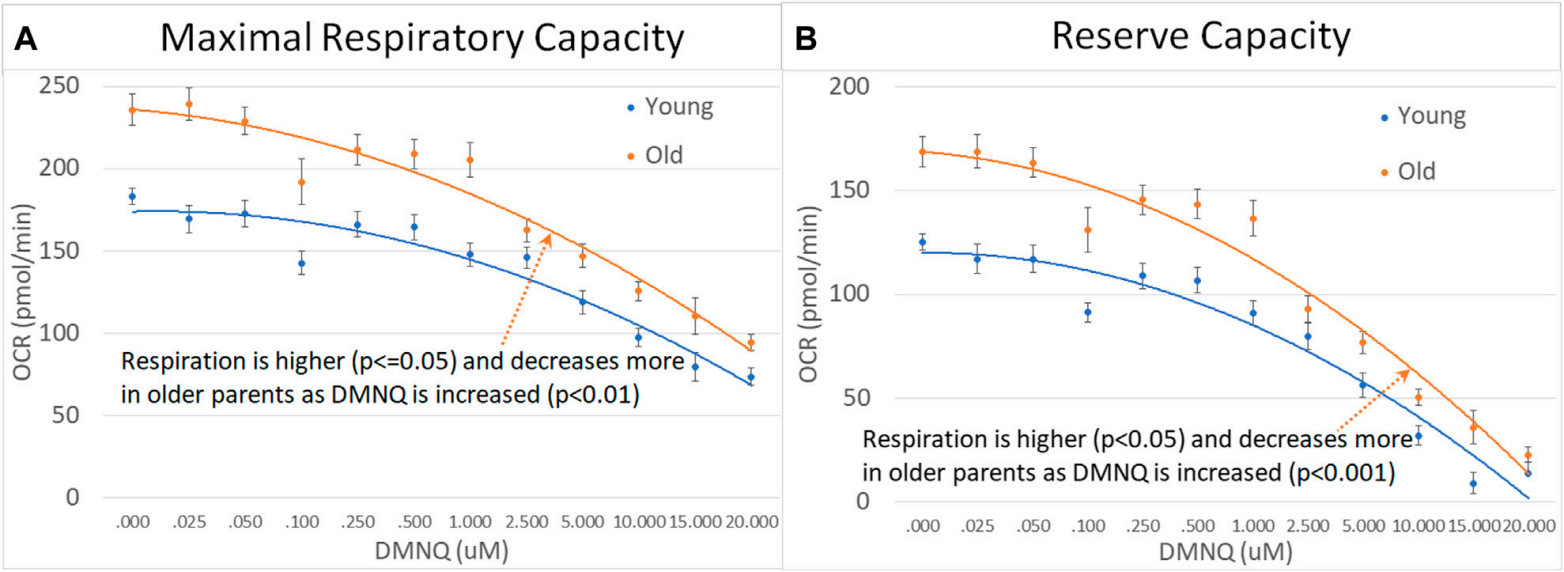
Parents of ASD children separated by age across mitochondrial respiratory parameters [measured by oxygen consumption rate (OCR)] with various amounts of physiological stress using increasing concentrations of 2,3-dimethoxy-1,4-napthoquinone (DMNQ). Standard error bars are shown.

We divided parents into age groups using the median age. Older parents were found to have higher MRC and RC as compared to younger parents with MRC and RC declining to a greater extent with increased DMNQ in older parents as compared to younger parents (Figure 8).

## 4 Discussion

In this study we extend our model of mitochondrial dysfunction in ASD-NDR children. First, we verified findings from our initial PBMC study in a separate patient cohort. Similar to our previous study (Singh et al., 2020), we have found that ASD-NDR individuals show elevated respiratory rates in their PBMCs as compared to ASD-NoNDR children. Second, we have extended the PBMC Seahorse assay using the MOST to investigate the changes in mitochondrial respiration in PBMCs as physiological stress is systemically added. This uncovered differences in the dynamics of mitochondrial respiration between ASD-NDR and ASD-NoNDR, similar to our LCL model of mitochondrial dysfunction in ASD (Rose et al., 2014; Rose et al., 2015a). Third, we demonstrated that this pattern of elevated respiration in ASD-NDR children was not found in their siblings, similar to the results of our LCL sibling study (Rose et al., 2017). Fourth, we found that both mothers and fathers of ASD-NDR children demonstrate atypical mitochondrial respiratory patterns similar to their children, suggesting that abnormalities in mitochondrial function in those with ASD-NDR are likely driven by similar biological underpinnings as their parents. This suggests that the abnormalities uncovered are not specific to a mitochondrial inherited component and not intrinsic to the child’s maternally inherited mitochondria. A sex effect was found consistently throughout the analyses, suggesting sex dependent differences in mitochondrial respiration. Lastly, mitochondrial respiration appeared to also be related to parental age.

The findings from each group comparison are summarized in Table 3 for the discussion below. The findings that are consistent across all groups are shaded to highlight the common mitochondrial physiological changes common to all groups.

**TABLE 3:** Summary of findings.

		ASD NDR vs. NoNDR	ASD vs. Siblings	Parents NDR vs. NoNDR
ALR	Mean	NDR higher	NDR higher	NDR higher
	Slope	NoNDR Decreases More		
PLR	Mean		NoNDR Higher	
	Slope	NDR Increases More	NoNDR Increases More	
MRC	Mean	NDR higher	NDR higher	NDR higher
	Slope	NDR Decreases More	NDR Decreases More	NDR Decreases More
RC	Mean	NDR higher	NDR higher	NDR higher
	Slope	NDR Decreases More	NDR Decreases More	NDR Decreases More
GR	Mean	NDR higher	NoNDR Lower	
	Slope		TD NoNDR Increases More	
GRC	Mean	NDR higher	NDR higher	NDR higher
	Slope		TD NoNDR Decreases More	

Findings that are consistent across all groups are shaded.

### 4.1 ASD-NDR children have unique patterns of mitochondrial respiration

Overall, ALR, MRC, and RC were significantly higher in ASD-NDR individuals as compared to ASD-NoNDR individuals (Table 3). As physiological stress was added (i.e., DMNQ was increased), both MRC and RC showed greater decrease in ASD-NDR individuals as compared to ASD-NoNDR individuals. These findings are consistent with our previous PBMC study on mitochondrial respiration in ASD individuals with and without NDR. Our previous PBMC study found that ASD-NDR individuals demonstrated significantly higher MRC and RC as compared to ASD-NoNDR individuals (Singh et al., 2020). These findings are also consistent with our LCL model of mitochondrial dysfunction in ASD (Rose et al., 2014; Rose et al., 2015a; Frye et al., 2016; Frye et al., 2017; Rose et al., 2017; Rose et al., 2018a; Bennuri et al., 2019). In our LCL studies we found a subset of children with ASD (called AD-A) with unusually high ALR, MRC and RC respiratory rates. This overactivity of the mitochondria has also been reported in other studies on ASD. In individuals with ASD, mitochondrial overactivity has been found in ETC Complex I in muscle (Graf et al., 2000) and in ETC Complex IV in muscle (Frye and Naviaux, 2011), fresh frozen superior temporal gyrus (Palmieri et al., 2010), buccal tissue (Delhey et al., 2017) and LCLs (Hassan et al., 2021). Recently we have linked ETC Complex IV overactivity with changes in mitochondrial morphology in fibroblasts (Frye et al., 2021b).

Our previous investigations of mitochondrial respiration in ASD using LCLs used the MOST to examine the effects of physiological stress on mitochondrial respiration but our previous PBMC study did not. Our LCL studies consistently found a subset of children with ASD (called AD-A) with a precipitous decrease in MRC and RC when physiological stress was added. This pattern of mitochondrial dysfunction was seen in the PBMCs in ASD-NDR individuals in the current study, suggesting a parallel between the ASD-NDR PBMCs and the AD-A LCLs. We proposed that the AD-A LCL subgroup was a model of NDR since RC, a measure of mitochondrial health, became significantly decreased with physiological stress, while the other group of ASD LCLs (called AD-N) which had mitochondrial respiration similar to TD controls did not show this precipitous decrease in RC (Rose et al., 2014; Rose et al., 2015a). Since children with NDR, especially those with underlying mitochondrial disorders, precipitously lose skills frequently in association with physiological stressors, it is very possible that a precipitous decrease in mitochondrial function could be the etiology of the loss of skills since the brain is the most metabolically active organ in the body (Hyder et al., 2013). Thus, we believe that the AD-A subset of LCLs represents a model of NDR in ASD.

In our previous LCL studies we showed that AD-A LCLs have higher mitochondrial reactive oxygen species (mtROS) as well as a higher amount of Uncoupling Protein 2 (UCP2) as compared to AD-N LCLs (Rose et al., 2014). UCP2 is essential for controlling proton leak across the inner mitochondrial membrane in order to reduce mtROS. Other studies have found an increase in UCP2 gene expression in children with ASD (Rose et al., 2017; Bennuri et al., 2019). In addition, studies have found another proton leak protein, the adenine nucleotide translocator (ANT), associated with ASD (Vandewalle et al., 2013; Picard et al., 2014). Interestingly, increased proton leak has recently been found to be a key factor in mitochondrial dysfunction in the Fragile X syndrome mouse (Licznerski et al., 2020).This latter mouse study demonstrated that increased proton leak in the mitochondria was associated with atypical synaptic growth, and that correcting the leak restored synaptic growth. Thus, high mtROS and engagement of mechanisms in an attempt to control mtROS may be directly related to abnormal synaptic function. The findings from our study demonstrate that the ASD-NDR group showed a greater proton leak compared to the ASD-NoNDR group, and this increased to a greater extent with the addition of physiological stress.

A greater proton leak in the inner mitochondrial membrane of the ASD-NDR and AD-A could explain the higher respiratory rates in these groups. The leak of more protons through the inner membrane decreases the inner membrane gradient which in turn decreases ATP production at ETC Complex V. As a response to this decrease in energy production, the mitochondria may very well upregulate the ETC in an attempt to reestablish adequate ATP production. We have also demonstrated that the AD-A LCLs are different in their mitochondrial response to environmental agents associated with ASD including trichloroacetaldehyde hydrate (Frye et al., 2017) and the microbiome associated short-chain fatty-acids propionate (Frye et al., 2016) and butyrate (Rose et al., 2018a). Further research will be needed to investigate the dynamic of this subset of ASD with mitochondrial that appear to uniquely adapt to changes in the physiological environment.

### 4.2 ASD-NDR siblings have normal mitochondrial respiration

We found that both ASD-NDR and ASD-NoNDR individuals demonstrated mitochondrial respiration changes different than their TD siblings, albeit in different respiratory parameters (Table 3). The differences in respiration between the ASD-NoNDR individuals and their TD siblings were similar to our previous LCL studies which compare individuals with ASD and their TD siblings (Rose et al., 2017) as well as unrelated TD controls (Rose et al., 2014). Interestingly, in our previous LCL study, both the individuals with ASD and their siblings demonstrated a decreased glutathione redox ratio while the unrelated TD controls did not manifest glutathione redox abnormalities. This suggests that the LCLs from both the ASD and TD sibling groups were under similar increases in oxidative stress. However, only the ASD individuals demonstrated the abnormal mitochondrial function. Given the potential link between controlling mitochondrial oxidative stress and the atypical mitochondrial respiratory profiles of the AD-A and ASD-NDR, it is possible that the LCLs from the TD siblings were able to buffer the increased oxidative stress without affecting mitochondrial function. With this perspective, it may be that the mitochondria of the TD siblings are more resilience that their ASD siblings.

### 4.3 Parents of ASD-NDR children show similar abnormalities as their ASD-NDR children.

We investigated mitochondrial respiration in the parents of children with ASD to determine if either parent demonstrated mitochondrial respiration abnormalities. This investigation demonstrated that both the mothers and fathers of children with ASD-NDR have atypical mitochondrial respiratory profiles similar to their children (Table 3). This supports the notion that this mitochondrial respiratory profile is heritable and that it does not occur through maternal mitochondrial transmission.

The fact that both parents express this atypical mitochondrial profile might point to a common environmental exposure that might be driving the mitochondrial abnormalities. This is supported by several studies. For example, long-term changes in mitochondrial function in children with ASD have been found to be related to prenatal environmental exposures such as air pollution (Frye et al., 2021a) and concentrations of the essential nutrient metals Zn and Mn (Curtin et al., 2018; Frye et al., 2020). In the two studies that measured mitochondrial function, these maternal environmental abnormalities were related to changes in mitochondrial function in ASD-NDR individuals (Frye et al., 2020; Frye et al., 2021a). Higher concentrations of air pollution were found to produce a long-term increase in mitochondrial respiration in ASD-NDR individuals similar to the respiratory increases seen in the ASD-NDR group in this study. Interestingly, similar changes in mitochondrial respiration have been associated with mouse prenatal environmental exposure models of ASD. Specifically, prenatal exposure to inflammation as in the MIA mouse, a model of ASD induced by prenatal immune environmental stress (Naviaux et al., 2013), and prenatal exposure to toxins as in the maternal valproic acid exposure mouse model of ASD (Ahn et al., 2014), demonstrate increases in mitochondrial respiration.

Prenatal changes in mitochondrial function in the fetus resulting from prenatal exposures may be maternally transmitted through cord blood. Maternal air pollution exposure is related to ETC dysfunction from mitochondria derived from cord blood (You et al., 2024) and exposure to arsenic metabolites (Qiu et al., 2023) and essential metals (Bi et al., 2023) are associated with changes in cord blood and newborn mitochondrial DNA copy number. Interestingly, cord blood mitochondrial DNA copy number was found to be associated with perinatal outcomes, such as gestation age, birth weight, and umbilical cord length (Fukunaga and Ikeda, 2023). The same environmental exposure that the child experiences prenatally is also experienced by the mother, so the changes in cord blood could be a biomarker of similar exposures, rather than method for transmitting changes in mitochondrial metabolism. In most cases, both parents are exposed to a similar environment through the prenatal period, suggesting that the similar changes in mitochondrial function could be caused by similar environmental exposures.

The fact that TD siblings of the ASD-NDR individuals seem to be resilient to these changes in mitochondrial function despite demonstrating markers of higher oxidative stress suggest a genetic or epigenetic component in the development of the abnormal mitochondrial respiration associated with ASD-NDR. Twin studies pointing to Zn and Mn prenatal deficits in ASD twins (Curtin et al., 2018; Frye et al., 2020) and the fact that prenatal Zn and Mn concentrations are associated with abnormal mitochondrial function in ASD-NDR individuals (Frye et al., 2020) points to a role for superoxide dismutase in the development of these abnormalities in mitochondrial respiration.

### 4.4 Differences in mitochondrial respiration in males and females

For parents, we found that males demonstrated a greater decrease in both MRC and RC with an increase in physiological stress. Differences in mitochondrial function have been uncovered in males and females in several studies. In general, mitochondria in women tend to rely on fats rather than carbohydrates and have higher functional capacity and greater resistance to oxidative damage than mitochondria in men (Mauvais-Jarvis, 2024). Other studies suggest that women may be better at the regulation of the redox state, as well as mitochondrial function, due to differences in sex hormones (Silaidos et al., 2018; Sultanova et al., 2020; Allegra et al., 2023). Such a finding is consistent with female mitochondria being more resilient.

However, for children with ASD, females demonstrated a greater decrease in MRC and RC with physiological stress, a finding that was opposite of parents and much of the literature. This is an interesting finding which should be followed up in the future. Given that studies in healthy individuals find that mitochondria of females may be more resilient, the overrepresentation of males in ASD could be due to their vulnerable mitochondria. The fact that females with ASD have more vulnerable mitochondria than males with ASD may represent a selection bias due to the females with vulnerable mitochondria being more susceptible to development ASD.

### 4.5 Age effects

Interestingly, there was no age effect on mitochondrial respiration for children with ASD but there was in the parents. Older parents demonstrated higher MRC and RC respiration with a greater decrease in MRC and RC as physiological stress was added, suggesting that older parents may have more vulnerable mitochondria. Other studies have examined changes in mitochondrial respiration with age, although the older adults used were much older than the older group of parents in our study. One study found a decrease in ATP and ETC Complex IV activity in the PBMCs of older adults which did not correlate with markers of energy metabolism in the brain (Silaidos et al., 2023). While another study also found a decrease mitochondrial respiration in the monocytes of older adults, the study did find that the mitochondria of older adults showed better coupling efficiency (Sriwichaiin et al., 2023). Another study found that monocytes of older adults demonstrated higher respiration than younger adults with an increase in respiration with exposure to inflammatory stimulation (Yarbro and Pence, 2019).

### 4.6 Etiological implications

Despite ASD being highly heritable, inherited defects in single genes are rare (Schaefer et al., 2013) and most identified single gene mutations are *de novo* (O'Roak et al., 2014; Wang et al., 2020). Even when both chromosomal microarray and whole exome sequencing are used, the unbiased empirical clinical genetic evaluations demonstrate low yields (−16%) (Tammimies et al., 2015) and only 31% of the time do siblings with ASD have the same *de novo* single gene mutations (Yuen et al., 2015). A better understanding of how to explain this disparity between heritability and empirical rates of single gene defects is starting to be uncovered. For example, one large study of multiplex ASD families pointed to a complex genetic risk architecture such that rare inherited protein-truncating variants in known ASD risk genes interacted with common gene variants result in a polygenic load which can be sufficient to cause a disease once a threshold is reached (Cirnigliaro et al., 2023). Furthermore, studies have demonstrated that mitochondrial haplogroups and polymorphisms are related to ASD risk (Chang et al., 2023). Thus, the search for single gene mutations in individuals with ASD oversimplifies the complex polygenic interactions that contribute to developing ASD.

However, even with better understanding of complex genetics, twin studies have shown that genetic variation only accounts for 38% of heritability, while shared environment accounts for approximately 58%, suggesting that the etiology of ASD may mainly be driven by genetic-environmental interactions (Hallmayer et al., 2011). Indeed, ASD risk can be linked to the maternal environment (Frye et al., 2021c; Frye et al., 2021d) and exposure to environmental toxins (Rossignol et al., 2014). Consistent with this notion, many physiological systems which are sensitive to environmental stressors have repeatedly been shown to be abnormal in children with ASD including the immune system (Hughes et al., 2023), transsulfuration (Howsmon et al., 2017), and mitochondrial metabolism (Rose et al., 2018b). This suggests that physiological disturbances in these pathways many be important in the etiology and ongoing disease in children with ASD, and may be pivotal biological systems in which the environment can transmit effects to increase the risk of ASD.

### 4.7 Clinical Implications

Although preliminary, these findings may have important clinical relevance. First, it is important to screen ASD-NDR children for abnormalities in mitochondrial function so the underlying abnormality can be minimized. Providing mitochondrial support may improve function and prevent further NDR episodes in these children in the future. It may also be important to screen siblings of ASD-NDR children early in life to determine if they exhibit any abnormalities in mitochondrial function so such abnormalities can be addressed, potentially preventing an NDR event. Lastly, the potential link between these mitochondrial respiratory abnormalities and environmental factors reinforces our knowledge that care should be taken during pregnancy to provide nutritional support and minimize stress.

### 4.8 Limitations

This study has several limitations. First, there were a limited number of siblings available for comparison. Further studies should increase the number of siblings to confirm these findings. Also, unlike our LCL model, RC for the ASD-NDR group did not eventually fall below the ASD-NDR group or TD siblings. This may be an artifact of the DMNQ concentrations used, as it appears higher concentrations of DMNQ are needed to decrease respiratory rates in the PBMCs as compared to the LCLs. The highest DMNQ concentration used was not sufficient to push RC below zero as it did in the LCL model. Indeed, future experiments should consider using higher DMNQ concentration with PBMCs. Given the sex and age effects, further studies should aim to match ASD-NDR and ASD-NoNDR participants on gender and age in order to optimally balance the investigation of this sex effect.

Additional assays would also be useful to complement the findings from the mitochondrial respiration measures that have been reported, particularly mitochondrial DNA copy number, mitochondrial content, measure of redox metabolism and key proteins and genes associated with regulation of the ETC and oxidative defenses.

## 5 Conclusion

This study provides further evidence that children with ASD and NDR may have a unique type of mitochondrial physiology that may make them susceptible to physiological stressors. Identifying these children early in life before NDR occurs and providing treatment to protect mitochondrial physiology could prevent NDR from occurring. The fact that siblings do not show these patterns is consistent with our previous LCL studies (Rose et al., 2017) and may imply that this physiological abnormality could have played a part in the etiology of their ASD. The finding that parents demonstrate mitochondrial respiration patterns similar to their children implies that this unique change in mitochondrial physiological may be a related to heritable factors (genetic or epigenetic), a result of shared environment, or both.

Most importantly, it would be important to determine if ASD-NDR children manifest these mitochondrial abnormalities from early in life before they undergo NDR. Early identification of such children may lead to preventative treatment that might prevent NDR. Importantly, this work also suggests that children with NDR may be more likely to have mitochondrial dysfunction, suggesting that it may be important to screen patients with ASD and NDR in the clinic for mitochondrial dysfunction and treat them accordingly.

## Data Availability

The raw data supporting the conclusion of this article will be made available by the authors, without undue reservation.
