# Supplementary material for: Bioenergetic signatures of neurodevelopmental regression
Source: Front Physiol. 2024 Feb 19;15:1306038. doi: 10.3389/fphys.2024.1306038 (PMC10916717; doi:10.3389/fphys.2024.1306038)
Supplement: Supplementary file 1 [file DataSheet1.docx]

| Supplemental Table 1. Statistical differences between NDR and NoNDR groups for children with ASD | | | |
| --- | --- | --- | --- |
|  | NDR vs NoNDR | | |
|  | Mean Difference | Linear Slope | Curved Slope |
| ALR | F(1,965) = 19.37*** | F(1,965) = 6.65** | F(1,965) = 4.68* |
| PLR |  | F(1,966) = 9.73** | F(1,966) = 4.48* |
| MRC | F(1,965) = 41.87*** | F(1,966) = 9.09** | F(1,965) = 4.30* |
| RC | F(1,966) = 35.40*** | F(1,965) = 11.99*** |  |
| GR | F(1,967) = 60.51*** |  |  |
| GRC | F(1,968) = 58.45*** |  |  |
| *p≤0.05, **p≤0.01, ***p≤0.001 | | | |

| Supplemental Table 2. Statistical differences between NDR and NoNDR groups and sex for children with ASD as compared to their sibling | | | |
| --- | --- | --- | --- |
|  | NDR vs NoNDR by TD vs ASD Interaction | | |
|  | Mean Difference | Linear Slope | Curved Slope |
| ALR | F(1,1115.5)=40.85*** | F(1,1108.1)=5.70** |  |
| PLR | F(1,1123.7)=15.25*** | F(1,1104.4)=12.41*** |  |
| MRC | F(1,1122.4)=65.41*** | F(1,1103.8)=8.78** |  |
| RC | F(1,1128.2)=72.41*** | F(1,1104.9)=18.34*** |  |
| GR | F(1,1113.6)=22.77*** | F(1,1102.1)=18.95*** | F(1,1102.1)=19.96*** |
| GRC | F(1,1009.8)=21.23*** | F(1,1106.0)=14.18*** | F(1,1106.0)=5.31* |
| *p≤0.05, **p≤0.01, ***p≤0.001 | | | |

| Supplemental Table 3. Statistical differences between NDR and NoNDR groups for parents of children with ASD | | | |
| --- | --- | --- | --- |
|  | NDR vs NoNDR | | |
|  | Mean Difference | Linear Slope | Curved Slope |
| ALR | F(1,44) = 16.69*** |  |  |
| MRC | F(1,42) = 6.60** | F(1,1275) = 10.17*** | F(1,1274) = 4.97* |
| RC | F(1,42) = 6.35* | F(1,1275) = 17.37*** | F(1,1275) = 9.24** |
| GR |  | F(1,1278) = 10.39*** | F(1,1278) = 6.39** |
| GRC | F(1,44.0) = 8.245** |  |  |
| *p≤0.05, **p≤0.01, ***p≤0.001 | | | |

| Supplemental Table 4. Statistical differences for sex for children with ASD | | | |
| --- | --- | --- | --- |
|  | Mean Difference | Linear Slope | Curved Slope |
| MRC | F(1,965) = 132.91*** |  |  |
| RC | F(1,966) = 130.95*** |  |  |
| GR | F(1,967) = 26.10*** |  |  |
| GRC | F(1,968) = 49.07*** |  |  |
| *p≤0.05, **p≤0.01, ***p≤0.001 | |  |  |

| Supplemental Table 5. Statistical differences sex for parents of children with ASD | | | |
| --- | --- | --- | --- |
|  | Sex | | |
|  | Mean Difference | Linear Slope | Curved Slope |
| MRC |  | F(1,1275) = 13.76*** | F(1,1274) = 6.29** |
| RC |  | F(1,1275) = 28.56*** | F(1,1275) = 15.23*** |
| *p≤0.05, **p≤0.01, ***p≤0.001 | | | |

| Supplemental Table 6. Age at blood draw for parents of children with ASD | | |
| --- | --- | --- |
|  | Age | |
|  | Mean Difference | Linear Slope |
| MRC | F(1,41) = 4.58* | F(1,1276) = 9.64** |
| RC | F(1,42) = 4.31* | F(1,1276) = 15.54*** |
| *p≤0.05, **p≤0.01, ***p≤0.001 | | |

| 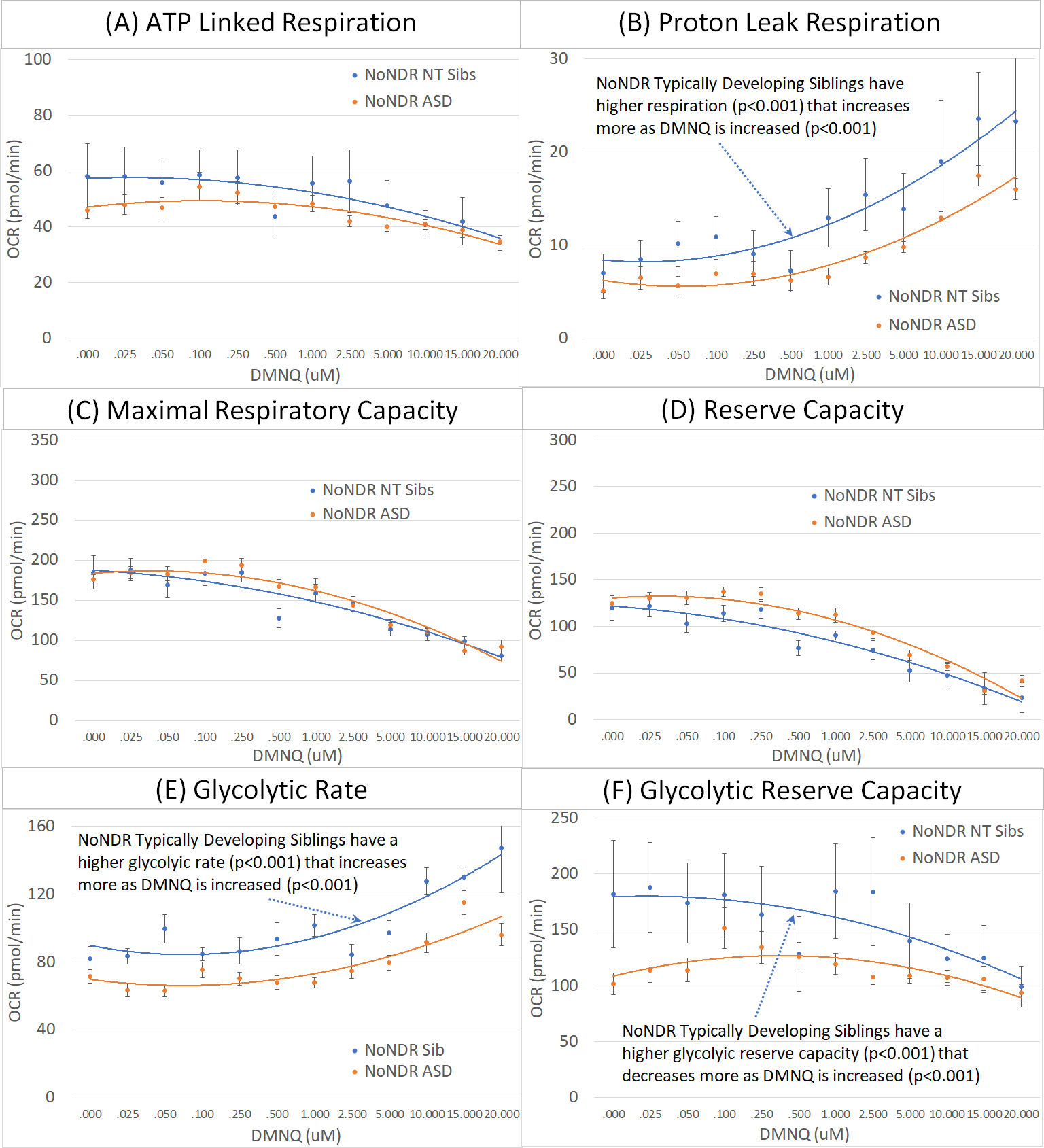 |
| --- |
| Supplementary Figure 1. |
